# Atomic Sulfur: An Element for Adaptation to an Oxidative Environment

**DOI:** 10.3390/molecules22111821

**Published:** 2017-10-26

**Authors:** Noryuki Nagahara, Maria Wróbel

**Affiliations:** 1 Isotope Research Center, Nippon Medical School, 1-1-5 Sendagi, Bunkyo-ku, 113-8602 Tokyo, Japan; 2 Chair of Medical Biochemistry, Kopernika 7 St., Jagiellonian University Medical College, 31-034 Kraków, Poland

During the period of rising oxygen concentration in the Earth’s atmosphere (Figure 1), sulfur atoms were incorporated into proteins as redox-active cysteine residues [1] and antioxidant molecules such as thioredoxin, glutathione, and glutaredoxin appeared. 

Cysteine residues in proteins form intra- and inter-molecular disulfides that maintain protein and peptide structure and also regulate protein function. In addition, the redox-active cysteine residues are known to regulate the redox state of proteins and function by reversible oxidation to cysteine sulfenic or cysteine sulfinic residues, or by -SH group sulfuration to persulfides. Finally, cysteine residues of the catalytic sites of such enzymes as sulfurtransferases and sulfotransferases, contributing to the transfer of elemental sulfur and sulfenate, respectively, are involved in sulfur metabolism. Recently it has been reported that four sulfurtransferases, cystathionine β-synthase (EC 4.2.1.22, CBS) [2], cystathionine γ-lyase (EC 4.4.1.1, CSE) [3], thiosulfate sulfurtransferase (rhodanese, TST) [4,5], and 3-mercaptopyruvate sulfurtransferase (MST) [4,5,6,7,8,9] produce hydrogen sulfide and polysulfides. The physiological functions of these sulfurtransferases have been elucidated.

Two years after the publication of the first special issue in 2014, a large number of investigations have demonstrated the extensive regulatory involvement of the endogenous pool of sulfide and, to date, research in this area has progressed significantly. In this issue, we present the findings and review work related to hydrogen sulfide and polysulfide, hydrogen sulfide and polysulfide-producing enzymes, antioxidative function and antioxidants (thioredoxin, glutathione, enzymes), cysteine persulfide and protein polysulfidation, sulfane sulfur, elemental sulfur, sulfur amino acids, hydrosulfide and tyrosine sulfation, including that from previous special issues.

The physiological actions of hydrogen sulfide and polysulfides have been widely reported [10,11,12,13,14,15,16]. Kimura reviewed the functions of H_2_S and polysulfides as biological mediators [10]. The concentration of H_2_S, determined by the activity of enzymes involved in its formation and clearance, depends on *S*-adenosyl methionine and CBS glutathionylation (both enhance the activity of CBS), NO and CO levels (suppress CBS activity), and Ca^2+^ concentrations (regulation of CSE and cysteine aminotransferase activity). However, the regulation of H_2_S degrading enzymes (Sulfide-Quinone Reductase and sulfur dioxygenase) is currently poorly understood. Polysulfides, found in tissues, modify the activities of channels, enzymes, and transcription factors through the mechanism of sulfuration. Polysulfide production, degradation, and regulation of these processes remain to be fully investigated and understood. The reaction of H_2_S with NO can result in the formation of highly reactive substances i.e., HSNO, GSNO, HNO, or HSSNO; elucidation of the mechanisms controlling their production and physiological effects will help to unravel the multiple roles of H_2_S and related molecules.

Hydrogen sulfide is synthesized in adipose tissue and is involved in the regulation of adipogenesis [11]. Deficiency of H_2_S may contribute to adipose tissue inflammation associated with obesity/metabolic syndrome. Experimental obesity induced by high calorie diets results in increased or decreased H_2_S in perivascular adipose tissue depending on duration time. Hyperglycemia suppresses the CSE-H_2_S pathway in various adipose tissue depots. Hence, it was proposed that augmentation of H_2_S signaling could diminish adipose tissue inflammation and correct related metabolic abnormalities.

Gastroprotective properties of H_2_S and its role in the defense mechanism against stress were confirmed by stimulation of H_2_S production and the observed beneficial effect of H_2_S on healing of gastric ulcers [12]. The results presented point to the possible role of rhodanese in H_2_S production in the gastric mucosa of rats.

The mechanism of H_2_S-mediated gastroprotection against ischemia/reperfusion (I/R) lesions has been studied [13]. I/R-induced gastric mucosa damage was reduced by pretreatment with H_2_S precursors (l-cysteine, GYY4137, NaHS) and this effect was accompanied by restoration of gastric microcirculation due to the vasodilatory activity of H_2_S. The mechanism was shown to involve the antioxidative properties of H_2_S, in the sense of increased expression of antioxidative enzymes (SOD-2 and GPx-1) in response to pretreatment with NaHS.

H_2_S produced in vascular smooth muscle cells can directly regulate the vascular tone in an autocrine manner. H_2_S synthesized in endothelial cells may have specific roles and specific targets e.g., Ca^2+^-activated K^+^ channels, endothelial NO synthase, and may be regulated independently of smooth muscle cells i.e., by mediators such as acetylcholine or leptin. In some pathological conditions, including obesity and I/R injury, endothelial H_2_S signaling is upregulated, which can be regarded as a specific protective mechanism, maintaining the regulation of vascular tone [14]. Challenges for future research include the regulation of endothelial H_2_S generation in pathological conditions, and possible therapeutic approaches to modulate its synthesis, metabolism, and signaling.

H_2_S is one among a number of molecules produced by the gut flora which may affect the circulatory system [15]. H_2_S released in the colon (sulfate reducing bacteria are ubiquitous in mammalian colon) may contribute to the control of arterial blood pressure. Studies suggest the possibility of modifying the flora for therapeutic benefit.

H_2_S and sulfane sulfur are formed from lipoic acid (LA) non-enzymatically but LA cannot be a direct donor of H_2_S/sulfane sulfur in tissues [16]. Under physiological conditions, an increased level of H_2_S in samples containing LA and dihydrolipoic acid (DHLA) was accompanied by a decrease in sulfane sulfur level. This suggests that DHLA acts as a reducing agent that releases H_2_S from sulfane sulfur-containing compounds, indicating the possible mechanism of pharmacological action of LA.

As for hydrogen sulfide and polysulfide related enzymes, the tissue and cellular distribution of MST in the mouse was further investigated by Tomita and colleagues [17]. Interestingly MST was extensively distributed in the endocrine organs of the mouse in addition to other tissues. They also confirmed the findings in rat tissues that Nagahara and colleagues previously reported [18]. Most and Papenbrock [19] reviewed the TST family of plant enzymes, which were shown to protect against cyanide toxicity and oxidative stress.

In relation to the antioxidative functions of sulfur, Mukwevho and colleagues [20] reviewed its integration into proteins as the redox-active cysteine residue and in species such as glutathione, thioredoxin, and glutaredoxin, which served as antioxidant molecules. Guevara-Flores and colleagues [21] reviewed unique thiol-based antioxidant systems in invertebrate parasites to understand their antioxidant defense mechanisms. This information could help to design drugs targeting these organisms. Bhuiyan and colleagues [22] reported that natural organosulfur compounds served as antioxidants and chemo-sensitizers and were implicated in the in vitro inhibition of tumor cell proliferation through the induction of apoptosis. TST catalyzes the H_2_S release reaction from garlic organosulfur compounds and Cys/GSH mixed disulfide conjugates that were spontaneously produced. Thus, a water-soluble, glutathione-garlic extract, serving as a slowly releasing hydrogen sulfide precursor was postulated to have potential therapeutic applications. Lin and colleagues [23] reviewed the evidence that gram-negative bacteria principally prevent cysteine overoxidation via the formation of mixed protein disulfides with low molecular weight thiols such as glutathione and glutathionyl spermidine. Cysteine was identified as highly susceptible to reactive oxygen species. Campos-Acevedo and Rudiño-Piñera [24] reported the optimum energy to perform X-ray of a crystal structure with an interface disulfide bond. The interface disulfide bond of thioredoxin 1 from *Litopenaeus vannamei* was very stable (less susceptible to being reduced by X-rays).

Cysteine persulfide and protein polysulfidation were reviewed by Kasamatsu and colleagues [25] who concluded that reactive persulfide species were physiologically important. Species such as cysteine persulfide and glutathione persulfide had higher nucleophilicity than the parents, cysteine (Cys) and glutathione. These reactive species protected against oxidants and contributed to redox signaling regulation. Protein polysulfidation also protected against oxidants.

Toohey and Cooper [26] reviewed the nature of thiosulfoxide (sulfane) sulfur, the history of its regulatory role, its generation in biological systems, and its functions, including synthesis of cofactors such as molybdenum cofactor and iron-sulfur clusters, sulfuration of tRNA, modulation of enzyme activities, and regulation of the redox environment.

Sulfane sulfur, the reactive sulfur atom in a zero to divalent (0 to −2) oxidation state can be converted to hydrogen sulfide on reduction with thiol-containing reducing agents [27]. Examples of this type of bound sulfur are the polysulfides involved in modification of protein function through persulfidation of cysteine residues (difficult to achieve with H_2_S directly), scavenging reactive carbonyl compounds in the central nervous system, and which also function in the redox regulation system in addition to other possible physiological roles.

Further studies [28] demonstrated high levels of sulfane sulfur (cyanolysable bound sulfur) in high grade gliomas (III/IV and IV grades) in comparison to various human brain regions which correlated with a decreased activity of CSE, MST, and TST. These results indicated the importance of sulfane sulfur for malignant cell proliferation and tumor growth and also suggest the possible application of dietary methionine restriction in the treatment of glioma. These observations suggest that further investigations should be carried out to confirm whether precursors of sulfane sulfur might inhibit the proliferation of gliomas. Sulfane sulfur dependency of the proliferation of cancer cells has been reported previously [29,30]. Opposing effects in malignant and normal cells opens up interesting opportunities for nutritional strategies in anticancer therapy.

In the case of the sulfur amino acids, excessive dietary intake of cystine and methionine can promote liver pathology such as diet-induced fatty liver [31]. The lipotropic effect of methionine may be mediated by sulfane sulfur. The hepatosteatogenic effect of cystine may be related to the removal of sulfane sulfur by cysteine catabolites. This form of sulfur is recognized as a regulatory agent in many physiological processes but the mechanism of control remains to be explained. Possible preventive and therapeutic strategies are discussed in relation to the avoidance of lifestyle induced health problems.

Toohey [32] has discussed the roles of hydrosulfide and hypothesized that the sulfur atom was involved in vitamin B_12_-dependent methyl group transfer. He further proposed that sulfane sulfur/hydrogen sulfide may be beneficial in treating Alzheimer’s disease. Finally, Yang and colleagues [33] reviewed the activity and function of tyrosylprotein sulfotransferase, which catalyzes the sulfation reaction, whereby sulfonate is transferred from 3′-phosphoadenosine-5′-phosphosulfate to tyrosine resulting in protein tyrosine sulfation and inducing protein-protein interactions.

## Figures and Tables

**Figure 1:**
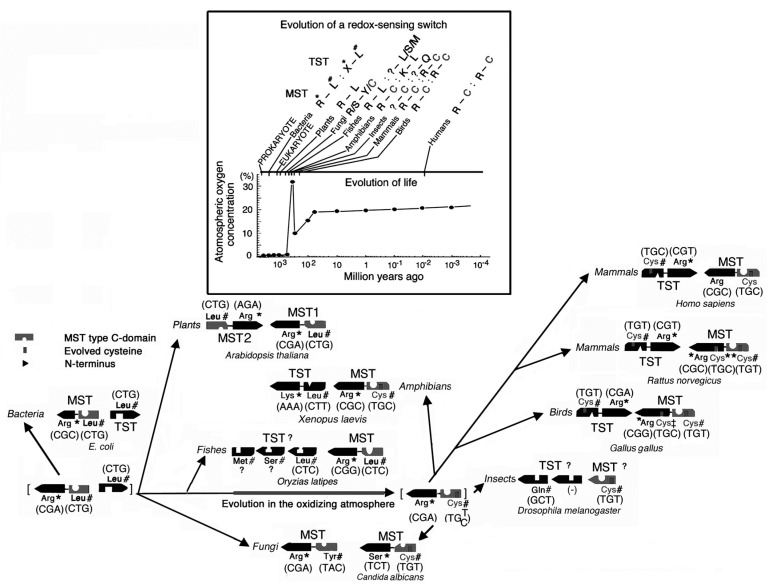
Example for molecular evolution of an intermolecular disulfide serving as a redox-sensing switch. In the box, molecular evolution of an intermolecular disulfide in a 3-mercaptopyruvate sulfurtransferase (MST) and rhodanese (TST) with changes in the Earth’s atmospheric oxygen concentration. Data for codons and deduced amino acid residues were based on cDNA or incomplete genomic DNA data: *Aspergillus oryzae* (AP007175 for MST); *Candida albicans* (XM_709437); *Drosophila melanogaster* (BLAST data form FlyBase; National Center for Biotechnology Information; and Berkeley Drosophila Genome Project); *Gallus gallus* (D50564 or XM_001231690 for MST, P25324 or XP_416284 for TST); *Oryzias latipes* (BLAST data form Medaka Expressed Sequence Tags data, the National Bio Resource Project Medaka Genome Project, and National Institute ofGenetics, DNA sequencing center); *Xenopus laevis* (BC08421 for MST and BC084422 for TST). C, Cysteine, which consists of a redox-sensing molecular switch; *, Amino acid corresponding to Arg^117^ of rat MST; #, Amino acid corresponding to Cys^247^ of rat MST (Source: Figure 10 from Nagahara [1]).
